# Meniscus of a Magnetic Fluid in the Field of a Current-Carrying Wire: Three-Dimensional Numerical Simulations

**DOI:** 10.3390/ma13030775

**Published:** 2020-02-08

**Authors:** Paul-Benjamin Eißman, Stefan Odenbach, Adrian Lange

**Affiliations:** Chair of Magnetofluiddynamics, Technische Universität Dresden, 01062 Dresden, Germany; paul_benjamin.eissmann@tu-dresden.de (P.-B.E.); stefan.odenbach@tu-dresden.de (S.O.)

**Keywords:** magnetic fluid, free surface instability, numerical simulations

## Abstract

Three-dimensional calculations of the meniscus of a magnetic fluid placed around a current carrying vertical and cylindrical wire are presented. Based on the material properties of experimentally used magnetic fluids, the numerically determined menisci are compared with the experimentally measured ones reported by May. The comparison is made for a linear law of magnetisation as well as for the experimentally measured nonlinear magnetisation curve. Up to moderate strengths of the applied current (I<=45 A), i.e., up to moderate strengths of the magnetic field close to the wire, the calculated profiles agree satisfyingly with the experimentally measured ones for a linear as well as for a nonlinear law of magnetisation. At a great strength of the applied current (I=70 A), i.e., at a large strength of the magnetic field close to the wire, the agreement is less good than in the range up to moderate strengths. Our analysis revealed that the numerically assumed isothermal conditions are not present in the experiment, particularly at the great strength of the applied current. A control of the temperature in the experiment and the implementation of a coupled thermal model in the numerics are considered the most relevant future steps for an improved agreement.

## 1. Introduction

One of the most fascinating characteristics of the physics of magnetic fluids (MFs) is the fact that the appearance of their free surface can be drastically modified under the influence of magnetic fields. The Rosensweig instability (see [1] and references therein) can be considered as an icon of such a behaviour, but also fluid layers subjected to nonuniform magnetic fields [2,3], fluid layers with cylindrical rods [4,5,6], singular drops [7,8,9,10,11,12] or sculptures [13] fashioned from these liquids exhibit in magnetic fields an astonishing variety and beauty in observable patterns and shapes.

A much simpler set-up to analyse is the meniscus of a horizontal layer of magnetic fluid in the field of a vertical current-carrying cylindrical wire [14]. An early analysis of that set-up is given in [15], where the influence of the surface tension was ignored, but leads to an analytical solution in this way. More than two decades later, the renewed interest started with a numerical work [16] on the resulting nonlinear differential equation describing the evolution of the surface as function of the distance from the wire and taking the surface tension into account. The derivation of this nonlinear differential equation is based on the assumption that isothermal conditions are present and uses the minimising of the total energy of the system. That total energy is the sum of three parts: the gravitational, the surface and the magnetic energy. Thus, the system is considered to be static, i.e., there is no flow field present. A transformation regularises the problem and grants a numerical solution. It followed a publication of experimental data [17] which allows a comparison with calculated results.

Our approach starts with the basic dynamic equation of hydrodynamics, the Navier–Stokes equation, extended to the case of a magnetic fluid in the presence of a magnetic field. The equation reduces to the nonlinear differential equation from [16] if an irrotational and stationary flow field is assumed. Therefore, dealing with that equation without assumptions forms a challenge. That is why the aim of this paper is to present the numerical simulation of the underlying Navier–Stokes equation without any assumption about the flow field for a three-dimensional configuration and compare the results with the experimental data.

## 2. Governing Equations

The starting point for deriving the governing equations is the time-dependent Bernoulli equation of a magnetic fluid (called medium I) in contact with a different medium II above [15] (Equation 5.5),

(1) $$\left(-\frac{\partial \phi }{\partial t}+\frac{v^{2}}{2}+g\xi \right)\rho +p-\mu_{0}\int_{0}^{H(\xi )}MdH^{\prime }=C^{\prime }\left(t\right)\;.$$

This equation has been derived under the assumptions that the MF is an incompressible Newtonian fluid, that magnetostriction is negligible, that the magnetisation M and the magnetic field H are collinear, that the flow field v is irrotational, i.e., rotv=0, and that the temperature in the entire system is constant. Due to the irrotational character of the flow field, it can be expressed by the corresponding velocity potential ϕ, v=−gradϕ. The two media are separated by an interface ξ(r,t), where r=(x,y) is perpendicular to the normal unit vector n, pointing from medium I to medium II. The pressure in the medium is denoted by *p*, ρ is the density of the MF, *g* the acceleration due to gravity, $\mu_{0}$ the permeability of free space and $C^{\prime }\left(t\right)$ a quantity solely depending on time *t*. As we are interested in the steady state of the static interface of the MF towards air as medium II, we assume that $v^{\left(II\right)}=M^{\left(II\right)}=\rho^{\left(II\right)}=v^{\left(I\right)}=0$ holds. Calculating now the difference between the Bernoulli equation of medium I and medium II, one gets

(2) $$\rho^{\left(I\right)}g\xi +p^{\left(I\right)}-\mu_{0}\int_{0}^{H^{\left(I\right)}\left(\xi \right)}M^{\left(I\right)}dH^{\prime (I)}-p^{\left(II\right)}=C^{\left(I\right)}-C^{\left(II\right)}=C\;,$$

where *C* denotes a constant since all dependencies on time dropped out. Equation (2) has to be supplemented by the boundary condition which states that the normal component of the stress tensor is continuous across the interface. This boundary condition reads finally as [15] (Equation 5.22)

(3) $$p^{\left(I\right)}-p^{\left(II\right)}=p_{c}-\frac{\mu_{0}}{2}{\left(M^{\left(I\right)}\cdot n\right)}^{2}+p_{0}^{\left(II\right)}\;,$$

where $p_{0}^{\left(II\right)}=p_{0}$ is the hydrostatic pressure on the nonmagnetic side and $p_{c}=\sigma K$ describes the capillary pressure with the surface tension σ and the curvature *K*. Substituting Equation (3) into Equation (2), the Bernoulli equation of a quiescent magnetic fluid with a free surface results (omitting the index I) in

(4) $$p_{0}+p_{c}+\rho g\xi -\mu_{0}\int_{0}^{H(\xi )}MdH^{\prime }-\frac{\mu_{0}}{2}M_{n}^{2}=C\;.$$

**Figure 1:**
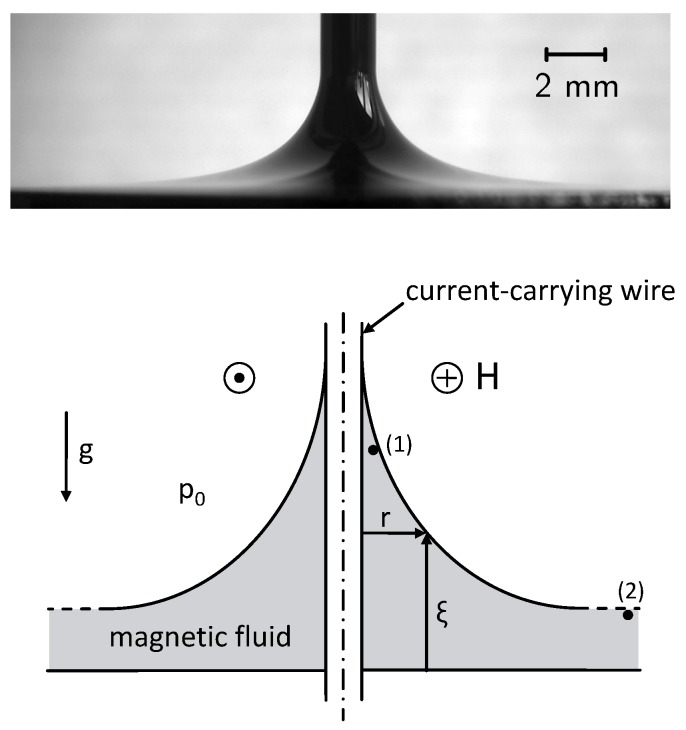
(**top**) Meniscus of the magnetic fluid EMG 909 around the wire carrying a current of 70 A. Courtesy of K. May [17]. (**bottom**) Sketch of the set-up (for details see text).

For the case of a vertical cylindrical current-carrying wire, considered here, the free surface forms a meniscus around the wire, as can be seen in the top part of Figure 1. In equilibrium all contributions of pressure at point (1), close to the wire, are equal to all contributions at point (2), far away from it, see bottom part of Figure 1. With the assumption that the free surface ξ(r) and the magnetic field $H=I/\left(2\pi r\right)e_{\varphi }$ of the wire tend to zero as *r* goes to infinity, Equation (4) reads

(5) $$p_{0}^{\left(1\right)}+p_{c}^{\left(1\right)}+\rho g\xi^{\left(1\right)}-\mu_{0}\int_{0}^{H(\xi^{\left(1\right)})}\;\;\;M\;dH^{\prime }-\frac{\mu_{0}}{2}{\left[M_{n}^{\left(1\right)}\right]}^{2}=p_{0}^{\left(2\right)}+p_{c}^{\left(2\right)}-\frac{\mu_{0}}{2}{\left[M_{n}^{\left(2\right)}\right]}^{2}\;,$$

where the current through the wire is denoted by *I*. The absolute value of the horizontal distance, r, the magnetic field as well as the magnetisation is given by r=|r|, H=|H|, M=|M| and $M_{n}=M\cdot n\;$, respectively. Equation (5) can be further reduced because the hydrostatic pressure is the same at both points and $p_{c}^{\left(2\right)}=0$ due to $K^{\left(2\right)}=0$. The latter results from the fact that for *r* to infinity the elevation of ξ diminishes. With a linear law of magnetisation, M=χH, and the feature that only the azimuthal component of the magnetic field is nonzero, one finally yields an analytical equation for the free surface of the magnetic fluid [16],

(6) $$\rho g\xi -\sigma \frac{\xi^{\prime }+\xi^{\prime 3}+r\xi^{\prime \prime }}{{\left(1+\xi^{\prime 2}\right)}^{3/2}}\frac{1}{r}-\frac{\mu_{0}\chi I^{2}}{8\pi^{2}}\frac{1}{r^{2}}=0\;,$$

where χ denotes the susceptibility of the MF and the primes the derivatives with respect to *r*.

**Figure 2:**
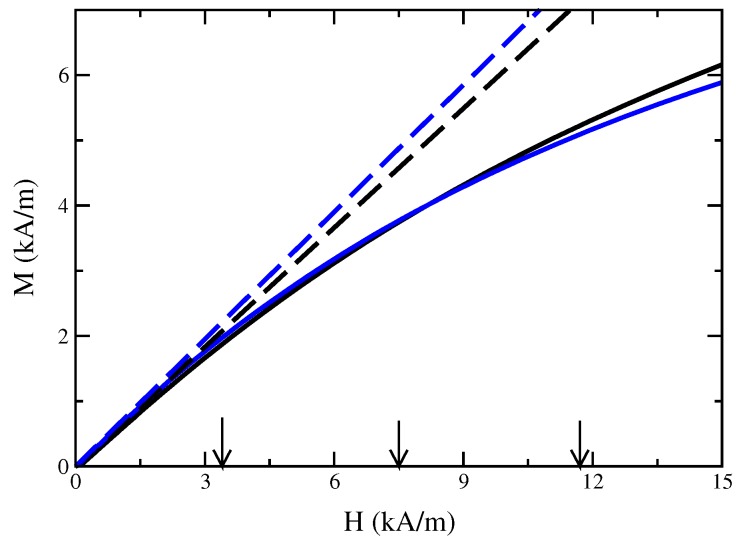
Experimentally measured magnetisation curve of EMG 909 (black solid line) and APG S21 (blue solid line), respectively, in the relevant range of *H*. Courtesy of K. May [17]. The black long-dashed (blue long-dashed) line indicates a linear law of magnetisation with χ=0.61 (χ=0.65) for EMG 909 (APG S21). The vertical arrows indicate the strength of the magnetic field at the rim of the wire at R=0.95 mm for I=20 A, 45 A and 70 A (from left to right).

An analytical solution for the simple case of σ=0 was already given in [15] (Chap. 5.4), but an analytical solution for the full problem (6) is not yet available. The reason is the nonlinear character of the equation caused by the nonlinear structure of the curvature in the term of capillary pressure. Therefore, John et al. in [16] proposed a transformation which regularises the problem and allows a numerical solution. Nevertheless, Equation (6) is an approximation since the derivation of the Bernoulli equation assumes isothermal conditions as well as an irrotational and stationary flow field. Therefore, the aim of this study is to present numerical simulations of the underlying Navier–Stokes equation [15] (Equation 5.2),

(7) $$\rho \left[\frac{\partial v}{\partial t}+\left(v\;\mathrm{grad}\right)v\right]=-\mathrm{grad}\;p+\eta \Delta v+\rho g+\mu_{0}\left(M\;\mathrm{grad}\right)H\;,$$

in three dimensions, where η denotes the dynamical viscosity of the magnetic fluid and g=(0,0,−g). No assumptions will be made about the flow field. The isothermal condition is kept for the simulations, but will be discussed separately in Section 4.2. In the three-dimensional *r*-φ-ξ coordinate system, the Kelvin force density is given by $f_{K}=\mu_{0}\left(M\;\mathrm{grad}\right)H=\mu_{0}M\left(\partial H/\partial r,0,0\right)$, where it was exploited that H and M are co-linear. For the simulations, either a linear law of magnetisation is used or the experimental data of the nonlinear behaviour of the magnetisation (Figure 2). The experiments were performed with the magnetic fluid EMG 909 (APG S21) having the material parameters ρ=1020 kg/m${}^{3}$ (1140 kg/m${}^{3}$), σ=0.0258 N/m (0.0331 N/m) and χ=0.61 (0.65) [17]. The dynamical viscosity is set to $\eta =10^{-3}$ kg/(m·s) since all tested values of η including the values given by the producer [18] lead to identical menisci [19]. The magnetic fluid EMG 909 (APG S21) is a light hydrocarbon oil-based (synthetic ester oil based) ferrofluid [18] with a saturation magnetisation of $M_{\mathrm{sat}}≃13.4$ kA/m ($M_{\mathrm{sat}}≃16.4$ kA/m) and particles with a mean diameter of $\bar{d}≃7.1$ nm ($\bar{d}≃7.0$ nm) [20]. The experimental set-up uses a wire with a radius of R = 0.95 mm and a welding transformer for the generation of the current which can achieve 100 Ampere in maximum [17]. Three different strengths (small, moderate and large) of the current were applied: I = 20 A, I = 45 A and I = 70 A.

## 3. Numerical Methods

**Figure 3:**
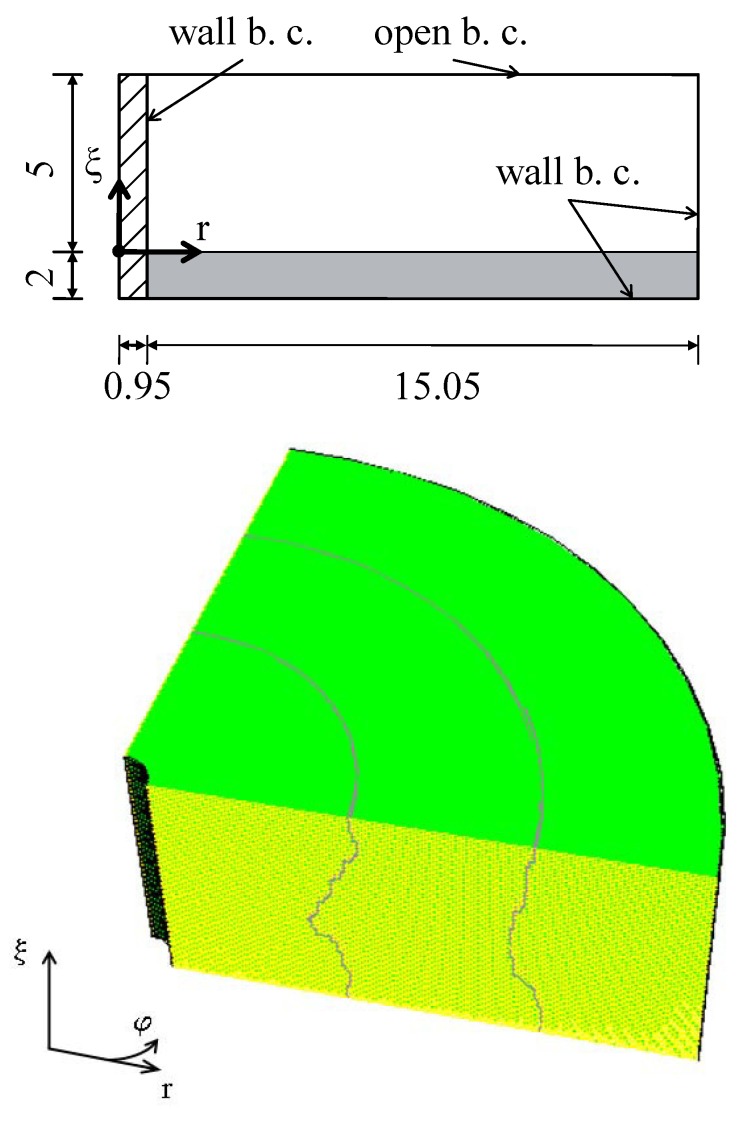
(**top**) Geometry and boundary conditions (b. c.) of the used numerical region (two-dimensional cross-section). The grey (shaded) region indicates the area of the magnetic fluid (wire). All measures are in millimetres. (**bottom**) Perspective view onto a quarter of the three-dimensional torus with an indication of its three subparts generated by FLUENT. The wall boundary conditions apply to the inner black curved area representing the rim of the wire, to the bottom of the torus (not visible in this perspective view), and to the outer black curved area (visible only as outer black line).

Extending the simulations in [19], the numerics here is done in three dimensions. The numerical calculations were conducted with the commercial software package ANSYS FLUENT 13.0 using the finite volume method for the two phases fluid and air. The domain of calculation is a torus of rectangular cross-section with a width of 16 mm and a height of 7 mm according to the experimental set-up in [17], where the origin is shifted by 2 mm in the vertical direction by the thickness of the layer of magnetic fluid, see Figure 3 (top). No-slip boundary conditions (wall b. c.) are imposed at all boundaries, with the exception of the upper horizontal boundary, where a so called open boundary condition is present. This condition allows an unconstrained flow of air in or out of the system. By using a finite volume method the numerical results are affected by the so called “flotsam” generated during the reconstruction of the phase boundary between the MF and air. The flotsam is considerably reduced by the use of FLUENT’s GEO-Reconstruct method which is based on Youngs’ technique [21]. That technique is superior to other approaches as a comparing study [22] shows. Only in the column nearest to the wire a few cells of flotsam remain. As beyond a simulation time of t=2.5 s in all tested cases this last flotsam does not move any more and does not influence the shape of the surface, it was removed. The change of the total mass of MF due to that removal can be neglected as the amount of flotsam is less than 0.05% of the total mass of fluid.

FLUENT’s numerical method for modelling the surface tension is based on the continuum surface force (CSF) model proposed in [23]. The CSF model uses the static angle of contact, established when the fluid is at rest. In agreement with this definition, the angle of contact of the MF with the wire was measured in [17,20]. By contrast, neither information about a change of this angle during the rise of the fluid at the wire nor any measurements of the angle of contact at the wall of the vessel are available [17,20]. Thus, the conducted numerics utilises static angles of contact only, where one of them is experimentally known.

To reduce the computing time only a quarter of the torus, supplemented with suitable conditions on the symmetry, was actually calculated. The area of calculation is divided in vertical direction into 50 cells, in radial direction into 480 cells and in azimutal direction at the outer rim into 250 cells. That sums totally up to approximately $2\times 10^{6}$ cells. That quarter was divided into three parts automatically by FLUENT, see Figure 3 (bottom), to allow a parallel calculation in each of them. The final computing time of a quarter of the torus summed up to two and a half days which corresponds to a real time of t=2.5 s.

The system calculated numerically here constitutes a model system with respect to geometry and the analytically known magnetic field. With such a model system, the quality of the numerics can be tested, particularly if experimental results are available. In that way, one receives knowledge about the strengths and weaknesses of the numerics of free surfaces of magnetic fluids which is helpful for the numerics of non-model systems as in the case of magnetic field concentrators [24] or locomotion systems [25].

## 4. Results

### 4.1. Menisci of the Magnetic Fluids

**Figure 4:**
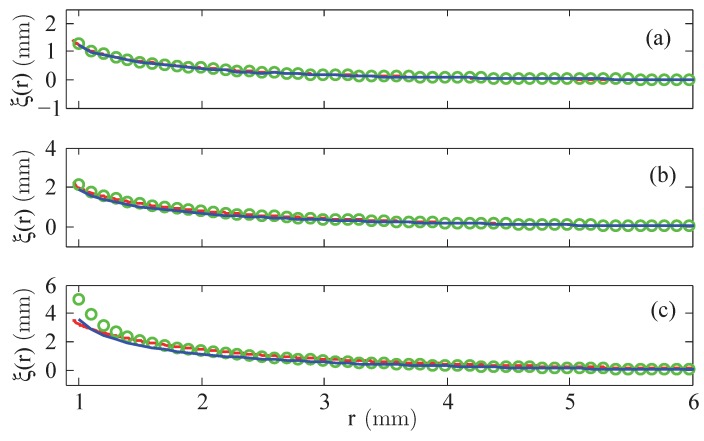
Calculated menisci of the magnetic fluid EMG 909 for a linear law of magnetisation with χ=0.61 (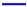) and χ=0.8 (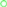) versus the experimentally determined surfaces (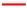). (**a**) H=3.35 kA/m, (**b**) H=7.54 kA/m, and (**c**) H=11.73 kA/m at R=0.95 mm.

The presentation of the results starts with the menisci of the fluid EMG 909, where, first, the numerical results using a linear law of magnetisation are compared with the experimental data. Figure 4 shows the shape of the final free surface for three different strengths (small, moderate and large) of the magnetic field, calculated at the rim of the wire at R=0.95 mm: H=3.35 kA/m (equivalent to I=20 A), H=7.54 kA/m (equivalent to I=45 A), and H=11.73 kA/m (equivalent to I=70 A).

**Figure 5:**
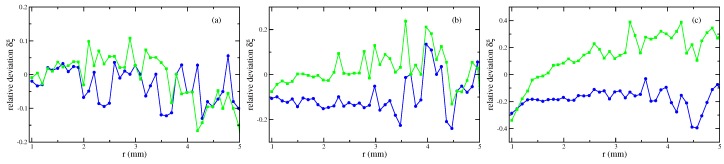
Relative deviation δξ, see Equation (8), of the numerically calculated menisci of the magnetic fluid EMG 909 using a linear law of magnetisation with χ=0.61 (•) and χ=0.8 (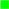). (**a**) H=3.35 kA/m, (**b**) H=7.54 kA/m and (**c**) H=11.73 kA/m at R=0.95 mm. The lines are guides for the eye. Note the different scales at the axes of ordinates.

For small magnetic fields, see Figure 4a, linear laws with χ=0.61 (blue solid line) but also with χ=0.8 (green circles), given by the producer [18], lead to a really good agreement with the experimental data. For an intermediate value of *H*, see Figure 4b, the graph generated by χ=0.8 gives a better agreement than the one by χ=0.61. At a high value of *H*, the calculated meniscus for χ=0.8 overestimates the real hight, whereas the meniscus for χ=0.61 slightly underestimates it. That analysis for all three strengths of the magnetic field is clearer visible by means of the relative error δξ, defined in Equation (8), which is plotted in Figure 5.

**Figure 6:**
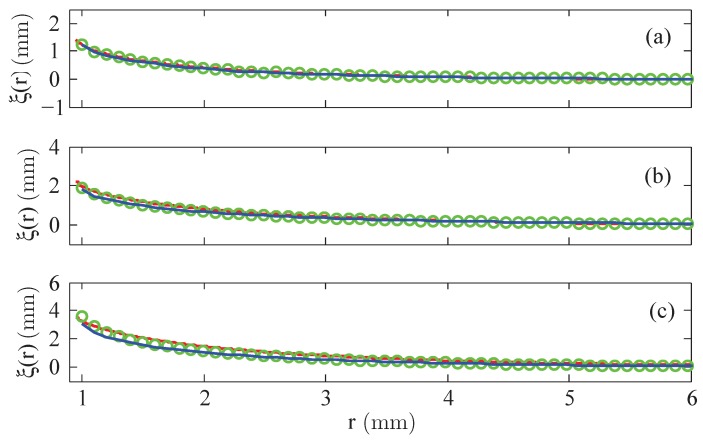
Calculated menisci of the magnetic fluid EMG 909 for a nonlinear law (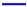) and linear law of magnetisation with χ=0.61 (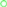) versus the experimentally determined surfaces (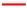). (**a**) H=3.35 kA/m, (**b**) H=7.54 kA/m and (**c**) H=11.73 kA/m at R=0.95 mm.

**Figure 7:**
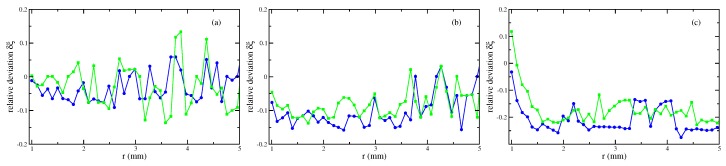
Relative deviation δξ, see Equation (8), of the numerically calculated menisci of the magnetic fluid EMG 909 using a nonlinear law of magnetisation (•) and a linear law with χ=0.61 (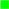). (**a**) H=3.35 kA/m, (**b**) H=7.54 kA/m and (**c**) H=11.73 kA/m at R=0.95 mm. The lines are guides for the eye. Note the different scales at the axes of ordinates.

Second, Figure 6 presents the results based upon the linear and the nonlinear law of magnetisation, respectively, both compared with the experimentally measured menisci. Up to intermediate values of the strength of the magnetic field, see Figure 6a,b, the agreement is fine; the maximal deviation numbers −12.2% (linear magnetisation) and −15.8% (nonlinear magnetisation), respectively, see Figure 7a,b. Only for the highest value of *H* in Figure 6c, both calculated curves differ from the experimental one, where the deviation is slightly larger for the nonlinear law (at most −25.8%) than for the linear one (at most −22.6%), see Figure 7c.

As summary of this set of results, one can say, that up to field strengths of ~7 kA/m, a linear law of magnetisation is a satisfying approximation, resulting in a good agreement with the experimentally measured menisci. For larger field strengths detectable deviations appear, where a linear law works better than a nonlinear one. An analysis regarding the reasons for this surprising outcome is presented in Section 4.2.

**Figure 8:**
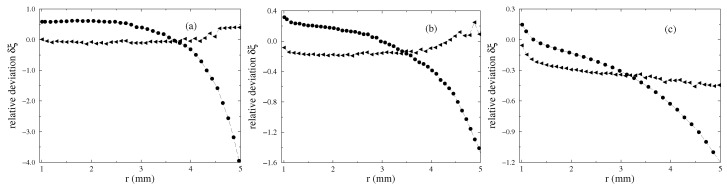
Relative deviation δξ using a nonlinear law of magnetisation in two-dimensional (• from [19]) and three-dimensional (◂) simulations, respectively: (**a**) H=3.35 kA/m, (**b**) H=7.54 kA/m and (**c**) H=11.73 kA/m at R=0.95 mm. The long dashed lines are guides for the eye. Note the different scales at the axes of ordinates.

One motivation to perform three-dimensional simulations is the expectation that three-dimensional numerics result in a better agreement with the experimental data. To confirm this expectation, the relative deviation with respect to the experimentally determined meniscus

(8) $$\delta \xi \left(r\right)=\frac{\xi_{\mathrm{num}}\left(r\right)-\xi_{\exp}\left(r\right)}{\xi_{\exp}\left(r\right)}$$

is calculated and plotted in Figure 8 for the three different strengths of the magnetic field using a nonlinear law of magnetisation. The upper limit of the considered range of *r* is determined by the fact that ξ(r≈5mm)≃0.2 mm for the largest strength of the magnetic field. Such an elevation is given in [20] as the error in the experimental determination of vertical elevations. The overall picture is that three-dimensional calculations generate a clear smaller relative deviation, i.e., the experimental menisci are better matched. For r>4 mm, the deviation of two-dimensional calculations [19] starts to become rather large. This is because these calculations are much less able to fashion tiny surface elevations compared to three-dimensional calculations. Those deviations increase for r>4 mm compared to the range 1mm≤r≤4mm [best seen in Figure 8a,b], but keep clear smaller than the corresponding deviations for two-dimensional calculations.

We point to a cautious handling of the numerical data with respect to the experimental ones. The absolute experimental error $\Delta \xi_{\exp}$ of the measured hight $\xi_{\exp}=\xi_{\exp}\left(r\right)$ of the surface is equal to $\Delta \xi_{\exp}=0.2$ mm over the entire range of *r* independent of the measured value of $\xi_{\exp}\left(r\right)$ [20]. (There are no information about the experimental error of $\xi_{\exp}\left(r\right)$ in [17].) Its leads to the situation that the relative error $\Delta \xi_{\exp}/\xi_{\exp}\left(r\right)$ reaches values of 1 and beyond for r≥2.9 mm at H=3.35 kA/m, for r≥4.0 mm at H=7.54 kA/m and for r≥5.4 mm at H=11.73 kA/m. That circumstance is clearly related to the experimental set-up and the resolution of the used camera in [20]. Nevertheless the experimental data of the surveyed surface are the only reasonable set of data the numerical ones can be compared with.

**Figure 9:**
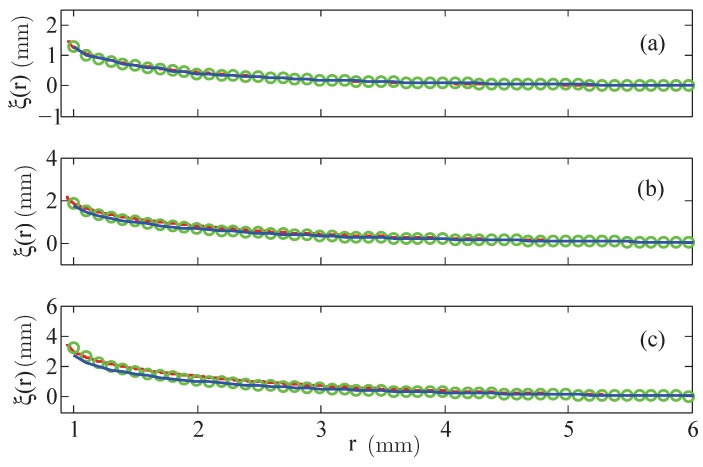
Calculated menisci of the magnetic fluid APG S21 for a nonlinear law (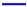) and linear law of magnetisation with χ=0.65 (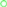) versus the experimentally determined surfaces (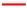). (**a**) H=3.35 kA/m, (**b**) H=7.54 kA/m and (**c**) H=11.73 kA/m at R=0.95 mm.

**Figure 10:**
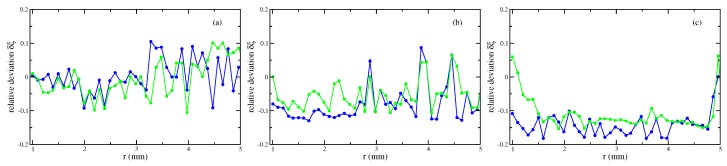
Relative deviation δξ, see Equation (8), of the numerically calculated menisci of the magnetic fluid APG S21 using a nonlinear law of magnetisation (•) and a linear law with χ=0.65 (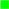). (**a**) H=3.35 kA/m, (**b**) H=7.54 kA/m and (**c**) H=11.73 kA/m at R=0.95 mm. The lines are guides for the eye.

The second set of results contains the numerically calculated menisci of the magnetic fluid APG S21, see Figure 9 and Figure 10. The reason for the test of the second MF, which has a similar density, surface tension and initial susceptibility as EMG 909, is that the rate of evaporation of APG S21 is considerably smaller than the one of EMG 909. That difference will come into play in the analysis in Section 4.2.

For a field strength of H=3.35 kA/m and H=7.54 kA/m, in Figure 9a,b, the calculated menisci agree rather well with the experimentally determined surfaces, where the maximal deviation amounts to −10.5% (linear magnetisation) and −12.1% (nonlinear magnetisation). The agreement becomes a bit inferior for the largest value of *H* with 11.73 kA/m, in Figure 9c, again the deviation is larger for the nonlinear law (maximal −18.0%) than for the linear one (maximal −15.3%).

For all calculated menisci of Figure 6 and Figure 9, it is noticeable that the numerical values underestimate the experimental data. In principle, higher calculated surfaces would appear for smaller angles of contact at the wire, but a test in [19] showed that the effect of angles smaller than the measured ones is negligible. Therefore, the assumption of isothermal condition deserves a consideration in more detail.

### 4.2. Temperature Distribution and Its Dynamics

To determine the temperature distribution in the magnetic fluid caused by the heat generation due to the current flowing through the wire, made out of copper, the three-dimensional torus with an even surface at ξ=2 mm is used. Besides the standard value for the density, $\rho_{\mathrm{Cu}}=8700$ kg/m${}^{3}$, the specific heat capacity, $c_{p,\mathrm{Cu}}=385$ J/(kg·K), the electrical conductivity, $\sigma_{\mathrm{ele},\mathrm{Cu}}=5.9\times 10^{7}$ V/(A·m) and the thermal conductivity, $\lambda_{\mathrm{Cu}}=400$ W/(m·K), of copper, one needs essentially the value of the thermal diffusivity κ of the tested fluid in order to solve the equation of heat conduction. Besides the density of the magnetic fluid, $\kappa =\lambda /(c_{p}\;\rho )$ involves its thermal conductivity and specific heat capacity. As no particular measurements of these quantities for EMG 909 are available, an estimation to $\kappa =7.5\times 10^{-8}$ m${}^{2}$/s from [26] was used. As boundary condition room temperature is assumed at the rim of the vessel. At the wire the temperature of the wire is given as boundary condition for the fluid. At t=0 the entire system has room temperature.

**Figure 11:**
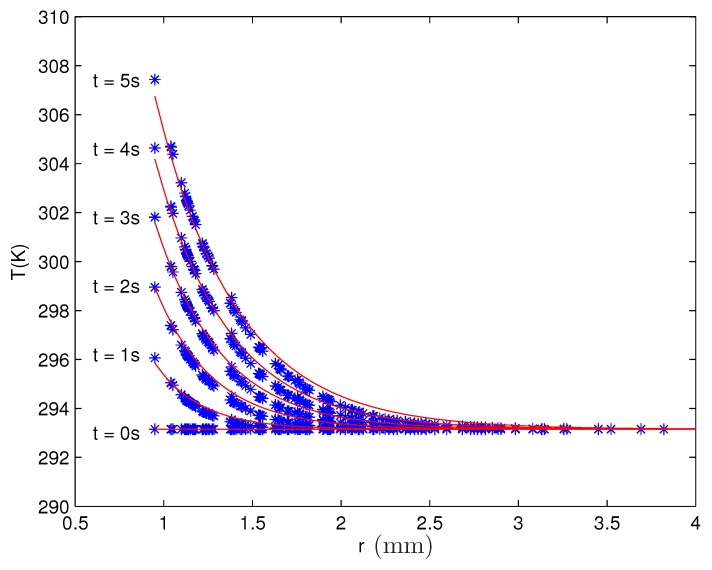
Radial distribution of the temperature of the magnetic fluid EMG 909 for a strength of the current of I=70 A applied over different spans of time. The blue stars (∗) denote the numerical results, and the red solid lines (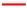) are exponential fits as a guide for the eye.

The radial distribution of temperature (the problem is axis-symmetric) shows Figure 11 for the case of the largest strength of the current, I=70 A, used in the experiments. The distribution towards the rim of the vessel follows an exponential decrease as the fits (see red lines) indicate. If one considers the temperature of the fluid just at the wire, it becomes clear that a significant increment happens. After an application of I=70 A over a span of time of t=5 s, the former room temperature of $20{\;}^{\circ }$C reaches nearly $34.3{\;}^{\circ }$C. Inspecting the case of t=2 s, the calculated temperature of $25.8{\;}^{\circ }$C is close to the temperature of $27{\;}^{\circ }$C measured with an infrared camera after 2 s in the experiment (see Figure 2 in [17]).

As at r≃3 mm (equivalent to approximately three times the radius of the wire) the entire increase of the temperature disappears, also significant gradients in the temperature are present. Altogether, the temperature itself and its gradient show that isothermal conditions are not present in a strict interpretation. Particularly, the surface tension, as a temperature-dependent quantity, will vary with time and in space contrary to the overall assumption that the surface tension is a constant.

To study the dynamics of the temperature distribution as a consequence of consecutive measurements with increasing strength of the current, the following procedure was implemented and simulated. Starting with an initial value of I=5 A, the current was increased step by step by ΔI=5 A up to the final value of I=70 A. Between each step a waiting time of Δt=300 s is engrafted modelling the experimental procedure described in [20]. The application of a waiting time aims for letting the fluid cool down again before the next strength of current is employed.

**Figure 12:**
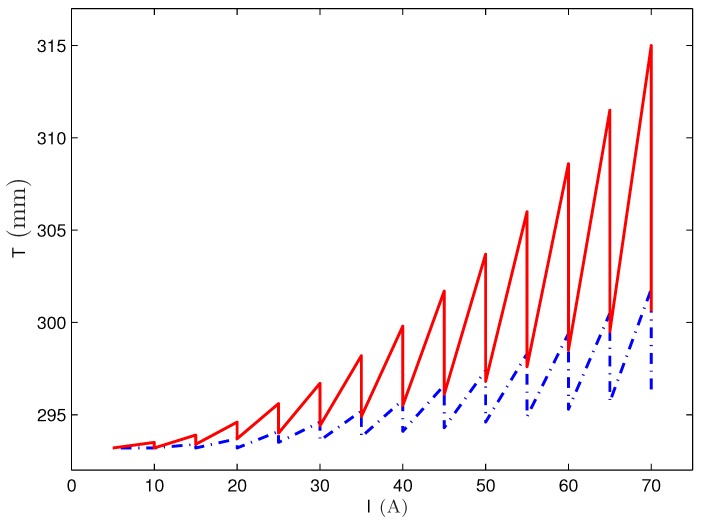
Maximal temperature of the ferrofluid EMG 909 for a consecutive increase of the current from I=5 A to I=70 A by ΔI=5 A. The waiting time between each increasing step counts to 300 s. The red solid line indicates the development of the temperature for a holding time of $\Delta t_{\mathrm{hold}}=5$ s at each strength of the current, and the blue dot-dashed one for $\Delta t_{\mathrm{hold}}=2$ s.

Figure 12 shows the development of the maximal temperature (present at the rim of the wire at R=0.95 mm) of the fluid for two different scenarios: in one case the holding time $\Delta t_{\mathrm{hold}}$ for each strength of current amounts to 5 s (red solid line), whereas the second case involves $\Delta t_{\mathrm{hold}}=2$ s (blue dot-dashed line). For the latter one, one can see that up to I=45 A, a waiting time of 300 s is enough that the fluid cools down to room temperature. Only beyond that strength of current, a slight but steady increase of the temperature during the waiting time is observable. For such a slight increase an evaporation rate of 9% per hour, as given by the producer for EMG 909 [18], there should not be a severe problem. The situation becomes different for $\Delta t_{\mathrm{hold}}=5$ s (red solid line). Already beyond I=25 A the cooling time of 5 min is not enough to reach room temperature. The final temperature at the end of the procedure is $∼39{\;}^{\circ }$C. Considering the total time over which the temperature of the fluid is now above room temperature, the given evaporation rate of EMG 909 poses a problem.

That conclusion is considered as a main reason for the larger deviation between the numerically calculated surface via a nonlinear law of magnetisation and the experimentally measured surface at a large strength of current. The higher temperature causes a certain evaporation of the carrier liquid, which increases the volume concentration of the magnetic nanoparticles during the experiments. Therefore, the measured nonlinear magnetisation curve *before* the experiments underestimates the magnetisation present *during* the experiments. By contrast, the linear law of magnetisation tends to higher values of *M* at the given strengths of the magnetic fields (see Figure 2), which is the reason why these deviations are smaller than the ones for a nonlinear law of magnetisation.

If one compares the results between the two fluids, the underestimation for APG S21 is approximately 25% smaller than the underestimation for EMG 909, valid for the linear and the nonlinear law of magnetisation, respectively. The cause for this reduced deviations results from the lower evaporation rate for APG S21. Thus the selection of a magnetic fluid with a rather low evaporation rate is desirable.

## 5. Discussion

In this section, the consequences of the absence of isothermal conditions are discussed in detail. An increase of the temperature, particularly close to the wire, will generate a raised evaporation. The resulting increase of the volume concentration of magnetic particles and the increase of the temperature itself cause different consequences for magnetisation and surface tension.

The strength of the magnetisation depends on two contrary dependencies: the increased volume concentration would increase the magnetisation, whereas the increase of the temperature would decrease the magnetisation; which dependency wins over the other is not clear, as measurements of the magnetisation as a function of the temperature, as in [27], did not determine in parallel the volume concentration. A similar behaviour applies to the density: an increase of the density by an increase in the volume concentration stays contrary to a decrease due to a higher temperature.

Furthermore, the surface tension is a critical factor in several respects, where two of them are related to changes of the temperature. First, an evaporation of the carrier liquid leads to a higher volume concentration of the magnetic particles which causes an increase of the surface tension as measured in [28]. Second, and simultaneously, a higher temperature brings forth a reduction of the surface tension. As no particular measurements of the temperature dependence of the surface tension of magnetic fluids are known, to the best knowledge of the authors, we are referring to recent measurements for binary mixtures [29], which confirm this dependence. With respect to magnetic fluids, it is an open question which of the two effects dominates the other one. Third, there is the size of the angle of contact at the wire. That angle is related to the surface tension between magnetic fluid and air by Young’s equation [30] expressing a necessary boundary condition for the fluid. Therefore a measurement of this angle during the experiments constitutes a vital information and would be an additional input for future numerical simulations, supported by the findings in [17] about the ferrofluid–air interface.

## 6. Summary and Future Work

Three-dimensional calculations of the meniscus of a magnetic fluid around a current carrying vertical and cylindrical wire are presented. Based on the material properties of experimentally used magnetic fluids, the numerically determined menisci are compared with the experimentally measured one. The comparison is made for a linear law of magnetisation as well as for the experimentally measured nonlinear magnetisation curve.

For a linear law of magnetisation, and for low as well as for moderate strengths of the current, the profiles agree satisfyingly up to 2 mm to the wire. Closer to the wire, and for high values of the current, the agreement is not so satisfying. Therefore, a nonlinear law of magnetisation was tested. The overall picture is that the maximal deviation for the nonlinear law of magnetisation is somehow larger than for the linear law. That is at first view counterintuitive since the nonlinear law of magnetisation is closer to the real properties of a magnetic fluid than a linear law. To resolve that puzzle, the thermal conditions in the experiment and in the simulations, respectively, come into play. For isothermal conditions, as present in the simulations, the linear law of magnetisation yields larger magnetisations than the nonlinear law with the exception of rather small magnetic fields. In the real experiment, the temperature is higher close to the wire than further away. An increase in the temperature causes two contrary dependencies: on the one hand, an increase of the magnetisation by an increased volume concentration of magnetic particles caused by evaporation, and on the other hand, a decrease of the magnetisation by the increased temperature itself. If the first dependency wins for the used magnetic fluids, the real magnetisation close to the wire is higher than the magnetisation further away, and higher magnetisations close to the wire generate a better agreement as the simulations with the less realistic linear law of magnetisation show. The fact that evaporation has a relevant influence on the results shows the better agreement for the fluid APG S21 with a lower evaporation rate in comparison with the fluid EMG 909.

The thermal aspects of the system turn out to be the most relevant ones in order to bridge the remaining gap between the real experiment and the model used for the calculations. A rising of the temperature modifies magnetisation as well as surface tension. In which way the modification takes place is not yet finally clarified. On the one hand, the increased temperature leads to a decrease of magnetisation and surface tension, whereas the increased volume concentration of magnetic particles, due to the raised evaporation, leads to an increase of both quantities. This situation directs the future work in several aspects.

From a numerical point of view, it would be desirable to implement a coupled thermal model since the present numerical model is limited to an isothermal situation. An essential requirement are reliable measurements of the dependence of the surface tension on temperature, as such measurements are not available presently. A parallel determination of the volume concentration of magnetic particles would deepen the understanding of the temperature effects. The same kind of measurements should be applied on the magnetisation. In this way, the magnetic liquids are suitable characterised if used for this type of experiments.

From an experimental point of view, a control of the temperature is considered essential. The aim should be to keep the temperature as best as possible constant, particularly in the vicinity of the wire. Therefore, a cooling of the wire as well as a tempering of the entire set-up would be advantageous in order to keep the differences in temperature as small as possible. To quantity the temperature control, measurements of the temperature of the wire and the fluid, the latter at different distances to the wire and different circumferential positions, should be implemented. Also, a measurement of the angle of contact of the magnetic fluid at the wire would add vital information about the behaviour of the surface at and close to the wire.

That all should lead to a better characterisation of the system and an improved agreement between experiment and numerics.

## References

[1] Richter R., Lange A., Odenbach S. (2009). Surface instabilites of ferrofluids. Collodial Magnetic Fluids: Bacisc, Development and Application of Ferrofluids.

[2] Becker T.I., Naletova V.A., Turkov V.A., Zimmermann K. (2017). Surface shape stability analysis of a magnetic fluid in the field of an electromagnet. J. Fluid Mech..

[3] Tenneti S., Subramanian S.G., ChakraBorty M., Soni G., DasGupta S. (2017). Magnetowetting of ferrofluidic thin liquid films. Sci. Rep..

[4] Pelevina D.A. (2016). Spape of the free surface of a magnetic fluid containing a cylindrical concentrator of the magnetic field. Fluid Dyn..

[5] Bashtovoi V., Motsar A., Naletova V., Reks A., Pelevina D. (2013). Free surface of magnetic fluid with spherical ferromagnetic body in a uniform magnetic field. Magnetohydrodynamics.

[6] Naletova V.A., Turkov V.A., Pelevina D.A., Rozin A.V., Zimmermann K., Popp J., Zeidis I. (2012). Behavior of a free surface of a magnetic fluid containing a magnetizable cylinder. J. Magn. Magn. Mat..

[7] Zhou J., Jing D. (2019). Effects of vertical magnetic field on impact dynamics of ferrofluid droplet onto a rigid substrate. Phys. Rev. Fluid..

[8] Ahmed A., Fleck B.A., Waghmare P.R. (2018). Maximum spreading of a ferrofluid droplet under the effect of magnetic field. Phys. Fluids.

[9] Ahmed A., Qureshi A.J., Fleck B.A., Waghmare P.R. (2018). Effects of magnetic field on the spreading dynamics of an impinging ferrofluid droplet. J. Colloid Interface Sci..

[10] Vinod S., Philip J. (2018). Field induced deformation of sessile ferrofluid droplets: effect of particle size distribution on magnetowetting. J. Magn. Magn. Mat..

[11] Rigone C., Pierno M., Mistura G., Talbot D., Massart R., Bacri J., Abou-Hassan A. (2016). Static magnetowetting of ferrofluid drops. Langmuir.

[12] Chen C., Li C. (2010). Ordered microdroplet formations of thin ferrofluid layer breakups. Phys. Fluids.

[13] Kodama S. (2008). Dynamic ferrofluid sculpture: Organic shape-changing art forms. Commun. ACM.

[14] Rosensweig R.E. (1987). Magnetic fluids. Ann. Rev. Fluid Mech..

[15] Rosensweig R.E. (1985). Ferrohydrodynamics.

[16] John T., Rannacher D., Engel A. (2007). Influence of surface tension of the conical meniscus of a magnetic fluid in the field of a current-carrying wire. J. Magn. Magn. Mat..

[17] John T., May M., Stannarius R. (2011). Meniscus of a ferrofluid around a vertical cylindrical wire carrying electrical current. Phys. Rev. E.

[18] Ferrotec Corp. www.ferrotec-europe.de/en/htmls/fluid.data.php.

[19] Eißmann P., Lange A., Odenbach S. (2011). Meniscus of a magnetic fluid in the field of a current-carrying wire: Two-dimensional numerical simulations. Magnetohydrodynamics.

[20] May K. (2010). Untersuchung des Meniskus von Ferrofluid an einem stromdurchflossenen Draht.

[21] Youngs D.L., Morton K.W., Baines M.J. (1982). Time-dependent multi-material flow with large fluid distortion. Numerical Methods for Fluid Dynamics.

[22] Rudman M. (1997). Volume-tracking methods for interfacial flow calculations. Int. J. Numer. Methods Fluids.

[23] Brackbill J.U., Kothe D.B., Zemach C. (1992). A continuum method for modeling surface tension. J. Comput. Phys..

[24] Naletova V.A., Pelevina D.A., Turkov V.A. (2009). Statics of a magnetic fluid containing magnetic field concentrators. Fluid Dyn..

[25] Zimmermann K., Zeidis I., Böhm V., Greiser S., Popp J. (2010). Ferrofluid-bases flow manipulation and locomation systems. J. Intell. Mater. Syst. Struct..

[26] Ludovisi D., Cha S.S., Ramachandran N., Worek W.M. (2007). Effect of magnetic field on thermocapillary and bouyancy driven flow of two immiscible liquids. 45th AIAA Aerospace Sciences Meeting and Exhibit.

[27] Virden A.E., O’Grady K. (2006). The temperature dependence of magnetisation in ferrofluids. J. Appl. Phys.

[28] Dababneh M.S., Ayoub N.Y., Odeh I., Laham N.M. (1993). Viscosity, resistivity and surface tension measurements of Fe_3_O_4_ ferrofluid. J. Magn. Magn. Mater..

[29] Ginder B., Villares A., Martin S., Artigas H., Lafuente C. (2007). Study of the temperature dependence of surface tensions of some alkanol + hexane mixtures. J. Chem. Eng. Data.

[30] Landau L.D., Lifshitz E.M. (1958). Statistical Physics.

